# CHARGE syndrome presenting with persistent hypoglycemia: case report and overview of the main genetic syndromes associated with neonatal hypoglycemia

**DOI:** 10.1186/s13052-022-01341-3

**Published:** 2022-08-20

**Authors:** Alessandra Consales, Beatrice Letizia Crippa, Lorenzo Colombo, Roberta Villa, Francesca Menni, Claudia Giavoli, Fabio Mosca, Maria Francesca Bedeschi

**Affiliations:** 1 Department of Clinical Sciences and Community Health, University of Milan, Milan, Italy; 2 Fondazione IRCCS Ca’ Granda Ospedale Maggiore Policlinico, NICU, Milan, Italy; 3 Clinical Genetics Unit, Fondazione IRCCS Ca’ Granda, Ospedale Maggiore Policlinico, Milan, Italy; 4 UOSD Pediatria Alta Intensità di Cura, Fondazione IRCCS Ca’ Granda Ospedale Maggiore Policlinico, Milan, Italy; 5 Endocrinology Unit, Fondazione Istituto di Ricerca e Cura a Carattere Scientifico (IRCCS) Ca’ Granda Ospedale Maggiore Policlinico, Milan, Italy

**Keywords:** neonatal hypoglycemia, CHARGE syndrome, case report, adrenal insufficiency, hyperinsulinemic hypoglycemia

## Abstract

**Background:**

CHARGE syndrome (CS) is an autosomal dominant genetic condition whose recognition in the neonatal period is complicated by considerable phenotypic variability. Pediatric patients with genetic disorders have a known high incidence of hypoglycemia, due to many concurring factors. To date, neonatal hypoglycemia is a feature poorly explored in the literature associated with CS. This paper adds to the existing literature on hypoglycemia in CS and provides a brief review of the mechanisms through which CS, as well as the main genetic syndromes associated with neonatal hypoglycemia, may determine it.

**Case presentation:**

The patient was a term newborn, first-born daughter to non-consanguineous parents. At birth, axial hypotonia with slight hypertonia of the limbs, and dysplastic auricles were noted. The incidental finding of asymptomatic hypoglycemia led to the initiation of glucose infusion on the II day of life, continued for a total of 8 days (maximum infusion rate: 8 mg/kg/min). In-depth endocrinological examinations showed poor cortisol response to the hypoglycemic stimulus, with normal GH values, thyroid function and ACTH. In view of the suspected hypoadrenalism, oral hydrocortisone therapy was initiated. Inappropriately low values of plasmatic and urinary ketones supported the hypothesis of concomitant transient hyperinsulinism, not requiring therapy. A brain MRI was performed, documenting thinning of the optic nerves, non-displayable olfactory bulbs and dysmorphic corpus callosum. An eye examination revealed bilateral chorioretinal coloboma. Temporal bone CT scan showed absence of the semicircular canals. The unexpected findings of coloboma and absence of semicircular canals led to the suspicion of CS, later confirmed by the molecular analysis of CHD7.

**Conclusions:**

It seems important to consider CS in the differential diagnosis of persistent hypoglycemia in newborns with specific anomalies. At the same time, it is advisable to consider the risk of hypoglycemia in children with CS, as well as other genetic syndromes. Awareness of the many possible causes of hypoglycemia in newborns with genetic conditions may help steer the investigations, allowing for an appropriate and timely treatment.

## Background

Hypoglycemia is a frequent metabolic finding in the neonatal period that can occur in up to 15% of newborns in the first days of life. Indeed, asymptomatic hypoglycemia in otherwise healthy newborns may be part of the physiologic transition to extrauterine life. However, caution is advised, since prolonged or recurrent hypoglycemia may lead to neurological damage and long-term developmental impairment. Newborns considered “at risk” for hypoglycemia, who therefore need special attention and adequate screening, include premature newborns, offspring of diabetic mothers, and newborns born large/small for gestational age. Several genetic syndromes are also known risk factors for the development of hypoglycemia during the neonatal period.

CHARGE syndrome (CS, OMIM #214800) is an autosomal dominant genetic condition with multisystem involvement and considerable phenotypic variability. Chromodomain helicase DNA binding protein 7 (CHD7) on chromosome 8q12.1 has been identified as the causative gene for CS. The cardinal features of CS are suggested by the acronym itself: Coloboma of the eye, Heart defects, choanal Atresia, Retardation of growth and/or development, Genito-urinary and Ear anomalies. Nonetheless, many other clinical features have been described over the years, identifying a spectrum of possible different presentations within the same condition. The diagnosis of CS is mainly clinical, based on the diagnostic criteria proposed by Blake et al. [1] in 1998 and updated in 2005 by Verloes [2]. Especially when dealing with a newborn, a high grade of suspicion is essential since a gestalt diagnosis may not always be feasible. Indeed, limited clinical recognition during the neonatal period has been linked to a high rate of underdiagnosis [3]. Consequently, genetic analysis plays a major role in the diagnosis of CS early in life [3], althoughonly approximately 60% of patients with clinical CS also have the corresponding genetic alteration [4].

We present the case of an unsuspected diagnosis of CS in a newborn whose first clinical finding was persistent asymptomatic hypoglycemia.

## Case presentation

Our patient, first-born daughter to healthy non-consanguineous parents, was delivered vaginally at 41 weeks of gestational age, after an uneventful pregnancy. The prenatal examinations initially raised the suspicion of an anomaly of the posterior cranial fossa, which was not confirmed by a level 2 ultrasound and neurosonography. At birth, slight hypertonia of the limbs and axial hypotonia were observed. Dysplastic auricles and a systolic 2/6 murmur were also noted. APGAR scores at 1 and 5 minutes were normal. Birth weight was 3706 g (appropriate for gestational age).

On day of life (doL) 1, the patient presented mild respiratory distress. Venous blood gas analysis thus performed showed borderline blood glucose levels (38 mg/dl at 2 hours of life), which persisted despite the initiation of enteral nutrition with formula milk. Although asymptomatic, the persistence of low blood glucose levels (lowest level: 28 mg/dl on doL 2) required the initiation of a 10% glucose intravenous infusion. The infusion was continued for a total of 8 days, with a maximum infusion rate of 8 mg/kg/min (10 mg/kg/min including enteral feedings). Subsequently, blood glucose levels were kept in range through frequent feedings. The in-depth endocrinological examinations performed showed poor cortisol response to the hypoglycemic stimulus (cortisol 7 ng/ml with glucose 28 mg/dl; expected value >18 ng/ml), normal ACTH (12.3 ng/L) and GH values (37.6 ng/ml during hypoglycemia; 9.38 ng/ml random), and adequate thyroid function, while gonadotropins and estrogens appeared inappropriately low for the minipuberty phase [5]. In view of the suspected hypoadrenalism, oral hydrocortisone therapy (7 mg/m^2^/die) was initiated at 1 week of life, with a subsequent progressive improvement of glucose profile. Second-level metabolic tests, including plasma aminoacids, urinary organic acids and plasma acylcarnitines did not suggest any metabolic issue. Inappropriately low plasmatic and urinary ketones, together with the fairly high glucose rate initially required to keep glycemic values in range, supported the hypothesis of transient hyperinsulinism, although not requiring pharmacological treatment and manageable with diet alone.

As part of the investigations for persistent neonatal hypoglycemia, in the suspicion of hypothalamic/pituitary abnormalities, an MRI was performed. The MRI documented numerous abnormalities: thinning of the optic nerves and the chiasm, non-displayable olfactory bulbs, and a dysmorphic appearance of corpus callosum, dentate nuclei and pons. Hypophyseal fossa and pituitary gland showed normal size and conformation.

The ophthalmological examination later showed bilateral chorioretinal coloboma.

The pathological cardiac auscultation (systolic murmur) and the ECG showing a right axis deviation warranted an echocardiography. The ultrasound examination showed a hemodynamically significant ostium secundum atrial septal defect and a patent ductus arteriosus. Oral therapy with furosemide was then initiated.

To better study the middle and inner ear in consideration of the dysplastic auricles, the newborn underwent a CT scan of the temporal bones. The CT scan showed multiple anomalies: hypoplastic stirrup, absence of the oval window and hypoplasia of the round window, absence of the semicircular canals, and hypoplasia of the medio-apical gyrus of the cochlea. Auditory evoked potentials demonstrated bilateral sensorineural hypoacusis.

Due to the presence of hypophonic cry, an otorhinolaryngology (ENT) consult was requested. Rhinolaryngoscopy showed hypotonia of the adductors of the vocal cords, and velopharyngeal sphincter.

The unexpected findings of coloboma of the eyes and absence of semicircular canals in a newborn with multiple specific anomalies sustained the suspicion of CS. The diagnosis was confirmed during the patient’s Neonatal Intensive Care Unit (NICU) stay by molecular analysis of the CHD7 gene, which showed a heterozygous non-sense mutation in exon 2 [c.1465 C>T (p.Gln489X)]. Sanger sequencing results showed that the mutation was not present in the patient’s parents, indicating that it was a *de novo* mutation.

The patient has been followed over the years at our Centre through multidisciplinary visits and periodic blood exams. The cardiac defects were surgically corrected at 14 months of age, with good surgical result. At 22 months of age, cochlear implant surgery was performed. At 3 years of age a low-dose ACTH stimulation test was programmed, but logistical problems limited its execution. However, ACTH and cortisol under venipuncture stress dosed in that occasion resulted normal (ACTH 22.9 ng/l, cortisol 19.9 mcg/dl), testifying the adequacy of the hypothalamic-pituitary-adrenal (HPA) axis. Hydrocortisone replacement therapy was therefore suspended. So far, blood exams have showed consistently normal glucose and hormonal values. The patient attends periodic ophthalmologic and ENT visits: no emerging issues have been highlighted. To date, the patient is well, with regular staturo-ponderal growth, and neurofunctional and psychomotor development.

## Discussion and conclusions

The diagnosis CS in the neonatal period is still a challenge for the neonatologist. Indeed, diagnosing CS in a newborn with multiple anomalies based solely on clinical findings is not an easy task. Genetic analysis is certainly a pivotal step in the diagnostic process this early in life, but a high level of suspicion is essential.

In the case we presented, integrated analysis of the clinical manifestations and results of examinations performed during our patient’s NICU stay suggested the diagnosis of CS, which was soon after confirmed by genetic testing. On retrospective evaluation, all the elements of the clinical picture fitted with a diagnosis of CS (Table 1), but the first diagnostic clue we had, that triggered all subsequent examinations, was rather non-specific: asymptomatic hypoglycemia. However, although a constitutive origin of the clinical picture presented by the patient could be hypothesized, hypoglycemia is a frequent finding in many genetic syndromes (Table 2). Conversely, it is a feature poorly explored in the literature associated with CS. Consequently, CS was not initially considered in the differential diagnosis of a patient with dysmorphic features and persistent hypoglycemia.

**Table 1:** Typical features of CHARGE syndrome and patient’s neonatal manifestations

Clinical features of CS	Patient’s neonatal manifestations
Ocular coloboma	Bilateral coloboma
Choanal atresia/stenosis	n/p
Cranial nerve dysfunction/anomaly	Agenesis of olfactory bulbs, thinning of optic nerves, bilateral sensorineural hypoacusis, vocal cord adductors and velopharyngeal sphincter hypotonia
Ear malformations	Dysplastic auricles, middle ear anomalies, absence of semicircular canals
Cleft lip and/or palate	n/p
Tracheoesophageal anomalies	n/p
Endocrine manifestations	Central hypoadrenalism, low gonadotropins and estrogens
Genital hypoplasia	n/p
Cardiovascular malformations	Ostium secundum ASD, PDA
Brain anomalies	Dysmorphic corpus callosum, cerebellum and pons
Renal anomalies	n/p
Developmental delay/intellectual disability	n/a

*Abbreviations*: *CS* CHARGE syndrome, *n/p* Not present, *n/a* Not applicable, *ASD* Atrial septal defect, *PDA* Patent ductus arteriosus.

**Table 2:** Overview of the main genetic syndromes that can present with neonatal hypoglycemia

Genetic Syndrome	Inheritance	Prevalence	Gene/Locus	Phenotype	Potential causes of HG	Incidence of HG
**Beckwith-Wiedemann**	AD	1:10,500-13,700	UPD 11p15	Macrosomia, macroglossia, hemihyperplasia, omphalocele, embryonal tumors, visceromegaly, adrenocortical cytomegaly, renal abnormalities, ear creases/pits	Hyperinsulinism	30-50%
**CHARGE**	AD	1**:**10,000-15,000	CHD7 8q12.2	Coloboma of the eye, heart defects, choanal atresia, growth retardation, genito-urinary and ear anomalies	Hyperinsulinism; GHD; feeding difficulties; CAI	n.a
**Costello**	AD	1:300,000-1:1.25M	HRAS 11p15.5	Coarse facial features (full lips, large mouth, full nasal tip); curly or sparse, fine hair; deep palmar and plantar creases; papillomata of the face and perianal region; ulnar deviation of the wrists and fingers; cardiac involvement	Hyperinsulinism; GHD; adrenal insufficiency	44%
**Donohue**	AR	<1:1M	INSR 19p13	Small, elfin-like face, protuberant ears, distended abdomen, acromegaly, hypertrichosis, acanthosis nigricans, decreased subcutaneous fat	Accelerated fasting state due to insulin resistance	n.a
**Kabuki**	KS1:AD KS2: XLR	1:32,000-1:86,000	KS1: KMT2D 12q13 KS2: KDM6A Xp11.3	Eversion of the lower lateral eyelid, depressed nasal tip, arched eyebrows, prominent ears; skeletal anomalies, abnormal dermatoglyphic presentation	Hyperinsulinism; GHD; CAI	6-8%, more common in KS2
**Laron**	AR	1-9:1M	GHR 5p13-12	Clinical hyposomatotropism, sparse hair, protruding and high forehead, shallow orbits, hypoplastic nasal bridge, small chin, hip degeneration	Resistance to GH counterregulatory activities	33-42%
**Perlman**	AR	<1:1M	DIS3L2 2q37.1	Hypotonia, inverted V-shaped upper lip, prominent forehead, deep-set eyes, broad and flat nasal bridge, low-set ears, renal anomalies	Hyperinsulinism due to Langerhans islets’ hypertrophy	n.a
**Prader-Willi**	AD	1:25,000	UDP 15q11–q13	Diminished fetal activity, obesity, muscular hypotonia, mental retardation, short stature, hypogonadotropic hypogonadism, small hands and feet	Adrenal insufficiency; GHD	12%
**Rabson-Mendenhall**	AR	n.a	INSR 19p13	Milder form of DS, hypertrichosis, acanthosis nigricans, prominent nipples, genital enlargement	Hyperinsulinism	n.a
**Silver Russell**	AD	1:30,000-1:100,000	SRS1: ICR1 11p15.5 SRS2: UPD cr. 7 SRS3: IGF2 11p15 SRS4: PLAG1 8q12 SRS5: HMGA2 12q14	Relative macrocephaly, triangular face, small lower jaw, low-set ears, frontal bossing, body asymmetry	Feeding difficulties; GHD; disproportionately large brain mass	27%-60%
**Simpson-Golabi-Behmel**	XLR	n.a	SGBS1: GPC3 Xq26 SGBS2: OFD1 Xp22	Coarse facies, large protruding jaw, widened nasal bridge, upturned nasal tip, enlarged tongue, congenital heart defects	Hyperinsulinism	n.a
**Sotos**	SoS1/2: AD SoS 3: AR	1:10,000-1:14,000	SoS1: NSD1 5q35 SoS2: NFIX 19p13 SoS3: APC2 19p13	Macrocephaly, dolichocephaly, broad and prominent forehead, sparse frontotemporal hair, downslanting palpebral fissures, long and narrow face	Hyperinsulinism	2-15%
**Timothy**	AD	<1:1M	CACNA1C 12p13	Hypotonia, round face, depressed nasal bridge, low-set ears, thin vermilion of the upper lip, hypoplastic premaxillary cutaneous syndactyly, congenital hip abnormalities, congenital heart disease, long QT syndrome	Hyperinsulinism due to altered influx of Ca^2+^ into pancreatic β-cells which triggers insulin secretion	36%
**Turner**	Sporadic	1-5:10,000 (1:2,500 F)	45, X0	Webbed neck, low hairline at the back of the neck, low-set ears, small mandible, lymphedema of extremities, multiple pigmented nevi	Hyperinsulinism due to increased sensitivity of pancreatic islets to aminoacids and elevated basal cytosolic calcium, possibly caused by haploinsufficiency of KDM6A (disruption of epigenetic changes during pancreatic differentiation); GHD	Rare

*Abbreviations*: *HG* Hypoglycemia, *n.a.* Data not available, *AR* Autosomal recessive, *AD* Autosomal dominant, *XLR* X-linked recessive, *UPD* Uniparental disomy, *GH* Growth hormone, *CAI* Central adrenal insufficiency, *M* Million, *KS* Kabuki syndrome, *ACTH* Adrenocorticotropic hormone, *SoS* Sotos syndrome, *DS* Donohue syndrome, *SRS* Silver Russell syndrome, *SGBS* Simpson-Golabi-Behmel, *F* Females.

Although lacking a definitive diagnosis, our patient’s hypoglycemia was later attributed to a combination of probable transient hyperinsulinism (self-limiting and not requiring pharmacological treatment) and suspected isolated central adrenal insufficiency (CAI), which justified the initiation of oral hydrocortisone therapy.

Hyperinsulinism is a feature frequently reported in many genetic syndromes, with varied etiologies. The most common overgrowth syndrome associated with hyperinsulinemic hypoglycemia (HH) is Beckwith-Wiedemann syndrome (BWS). BWS is a rather uncommon cause of neonatal HH, although the incidence of hypoglycemia in BWS is reported to be around 50%. Infants with BWS typically develop hypoglycemia within the first few days of life and usually respond well to oral therapy with diazoxide [6].

In CS, Sekiguchi et al. [7] hypothesized that hyperinsulinism might be the consequence of the altered expression of homeobox A5 in the fetal pancreas due to CHD7 mutations, which might impair the differentiation and/or function of β cells. Interestingly, homeobox A5 is also the target gene of KMT2D, the causative gene for Kabuki syndrome (KS). CS and KS are considered clinically overlapping conditions [8, 9]. Hypoglycemia, either transient or persistent, is a common feature of KS, and it has been variably associated with hyperinsulinism, GH deficiency (GHD) and isolated ACTH deficiency [10].

It has been suggested that CHD7 mutations may also lead to a continuum of anatomical and functional hypothalamic/pituitary abnormalities [11]. Pituitary hormone deficiency is a known feature of CS, most notably resulting in hypogonadotropic hypogonadism. Indeed, in our patient, the evaluation of the hypothalamic-pituitary-gonadal (HPG) axis represented an ulterior element that supported our clinical suspicion of CS. Conversely, CAI has seldom been reported [12] and it is currently not considered a common feature of CS [13]. In our patient, given the low cortisol levels and inappropriately normal ACTH values during severe hypoglycemia, and the prompt normalization of glucose profile after hydrocortisone replacement (which suggested an *ex juvantibus* diagnosis), we did not perform a specific stimulation test during the birth hospitalization, but we planned a dynamic evaluation of the HPA axis at an older age. The following finding of a normal adrenal function at 3 years of age does not exclude a possible initial immaturity of the HPA axis in the neonatal period.

Cortisol deficiency has been identified as a potential cause of hypoglycemia also in patients with Costello syndrome (CoS) [14], a RAS-opathy with multisystem involvement. Moreover, adrenal insufficiency has been found in up to 60% of patients with Prader-Willi syndrome (PWS) and it has been proposed as one of the probable causes of the hypoglycemia frequently presented by these patients.

In addition to adrenal insufficiency and HH, patients with CS may develop hypoglycemia due to another of the many endocrinological issues found in this syndrome (Table 3): GHD. A recent case report [19] showed how hypoglycemia in a patient with CS proved to be an early sign of GHD, allowing for a timely and successful treatment with recombinant GH, before growth retardation could be noted. GHD is a frequent finding in many other genetic syndromes, such as KS, CoS, PWS and Silver-Russell syndrome (SRS). In our patient, GH levels were adequate both during hypoglycemia and randomly, ruling out a GHD. Indeed, as indicated by recent guidelines, stimulation tests are not required in the neonatal period and a random GH level <7 ng/ml in the first week of life supports a biochemical diagnosis [20].

**Table 3:** Summary of endocrine conditions reported in patients with CHARGE syndrome

Condition	Clinical manifestations	Frequency	Management	Comment
Hypogonadotropic hypogonadism	Genital hypoplasia (micropenis, cryptorchidism, hypoplastic labia/clitoris), pubertal delay/arrest	50-70%	Testosterone/Oestrogen replacement therapy	HHG in association with anosmia overlaps with Kallmann syndrome [15]
Hypothyroidism	Jaundice, lethargy and feeding difficulties (newborn), failure to thrive	15-20%	Thyroxine replacement therapy	Routine biochemical assessment of thyroid function not necessary, reserved for patients with clinical features of hypothyroidism [16]
GH deficiency	Poor growth, short stature, hypoglycemia	9%	GH replacement therapy	Short stature in CS usually not solely due to hormone deficiency [17]
Secondary hypoadrenalism	Adrenal crisis with hypoglycemia and hyponatremia	n.a	Hydrocortisone	Not observed in large cohorts of subjects [12, 13]
Congenital hypopituitarism	Multiple pituitary hormone deficiencies	n.a	Hormone replacement therapy	Associated with structural anomalies of the anterior or posterior pituitary gland [11]
Hypoparathyroidism	Hypocalcemia	n.a.	Activated vitamin D (calcitriol) and calcium supplements	HP, also seen in association with immunological anomalies, overlaps with Di George syndrome [18]
Hyperinsulinemic hypoglycemia	Hypoglycemia	n.a	Diazoxide	Reported in a 2-year-old Japanese male patient by Sekiguchi et al. [7]

*Abbreviations*: *CS* CHARGE syndrome, *HHG* Hypogonadotropic hypogonadism, *GH* Growth hormone, *n.a.* Data not available, *HP* Hypoparathyroidism.

Finally, in CS, hypoglycemia could be a result of the known feeding difficulties many patients experience [21]. Several features typical of CS contribute to the feeding difficulties reported in around 100% of patients. Cranial nerve dysfunction understandably plays a major role, especially determining facial palsy and swallowing difficulties. However, cleft lip/palate, choanal atresia, cardiac structural defects and tracheoesophageal anomalies may give a substantial contribution as well. Feeding problems are common among patients with genetic syndromes. For instance, in patients with SRS, reduced caloric intake due to feeding difficulties including poor appetite, slow feeding and oral-motor dysfunction, has been reported as a possible cause of hypoglycemia [22].

In conclusion, although further studies are needed to evaluate the exact prevalence of hypoglycemia in patients with CS, we believe that it is important to consider this syndrome in the differential diagnosis of persistent hypoglycemia in newborns with specific anomalies. At the same time, it is advisable to consider the risk of hypoglycemia in children with CS, as well as other genetic syndromes. Frequent feedings and a careful blood glucose monitoring in patients with multiple anomalies, even before reaching a definitive diagnosis, may prevent episodes of hypoglycemia leading to permanent neurologic damage. Moreover, awareness of the many possible causes of hypoglycemia in newborns with CS, as well as other genetic syndromes, may help steer the investigations, allowing for an appropriate and timely treatment (e.g., diazoxide in case of HH, GH replacement therapy in GHD or hydrocortisone therapy in CAI).

## Data Availability

Not applicable.

## Abbreviations

ACTH: Adrenocorticotropic hormone
BWS: Beckwith-Wiedemann syndrome
CAI: Central adrenal insufficiency
CHD7: Chromodomain helicase DNA binding protein-7
CoS: Costello syndrome
CS: CHARGE syndrome
DoL: Day of life
ENT: Otorhinolaryngology
GH: Growth hormone
GHD: Growth hormone deficiency
HH: Hyperinsulinemic hypoglycemia
HPA: Hypothalamic-pituitary-adrenal (axis)
HPG: Hypothalamic-pituitary-gonadal (axis)
KS: Kabuki syndrome
PWS: Prader-Willi syndrome
SRS: Silver-Russell syndrome
